# Isogenic Cellular Systems Model the Impact of Genetic Risk Variants in the Pathogenesis of Type 1 Diabetes

**DOI:** 10.3389/fendo.2017.00276

**Published:** 2017-10-18

**Authors:** Mark A. Wallet, Katherine E. Santostefano, Naohiro Terada, Todd M. Brusko

**Affiliations:** ^1^Department of Pathology, Immunology, and Laboratory Medicine, University of Florida Diabetes Institute, College of Medicine, Gainesville, FL, United States

**Keywords:** type 1 diabetes, autoimmunity, induced pluripotent stem cells, gene editing, genome-wide association studies, expression quantitative trait loci

## Abstract

At least 57 independent loci within the human genome confer varying degrees of risk for the development of type 1 diabetes (T1D). The majority of these variants are thought to contribute to overall genetic risk by modulating host innate and adaptive immune responses, ultimately resulting in a loss of immunological tolerance to β cell antigens. Early efforts to link specific risk variants with functional alterations in host immune responses have employed animal models or genotype-selected individuals from clinical bioresource banks. While some notable genotype:phenotype associations have been described, there remains an urgent need to accelerate the discovery of causal variants and elucidate the molecular mechanisms by which susceptible alleles alter immune functions. One significant limitation has been the inability to study human T1D risk loci on an isogenic background. The advent of induced pluripotent stem cells (iPSCs) and genome-editing technologies have made it possible to address a number of these outstanding questions. Specifically, the ability to drive multiple cell fates from iPSC under isogenic conditions now facilitates the analysis of causal variants in multiple cellular lineages. Bioinformatic analyses have revealed that T1D risk genes cluster within a limited number of immune signaling pathways, yet the relevant immune cell subsets and cellular activation states in which candidate risk genes impact cellular activities remain largely unknown. In this review, we summarize the functional impact of several candidate risk variants on host immunity in T1D and present an isogenic disease-in-a-dish model system for interrogating risk variants, with the goal of expediting precision therapeutics in T1D.

## Introduction

**Figure 1:**
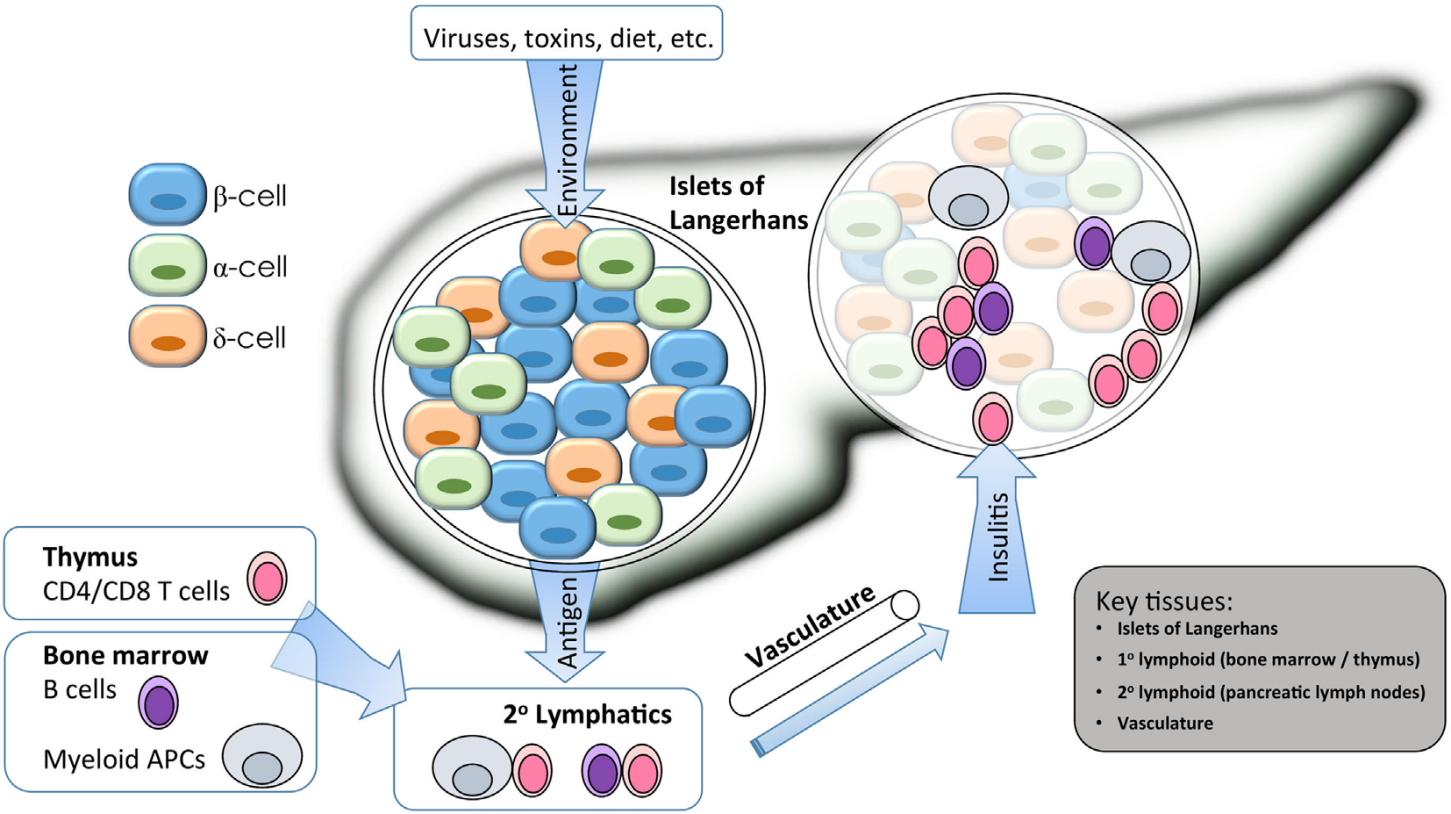
Isogenic modeling facilitates the investigation of multiple cell types important in the pathogenesis of type 1 diabetes (T1D). A combination of environmental and genetic factors influences the overall risk for T1D. Genes conferring risk for T1D may affect the functions of β cells, immune cells, and vascular endothelium. For β cells, risk variants of some genes may alter the response to environmental triggers such as inflammatory or viral sensing, or they may alter the way that β cells cope with stress from bioenergetic demands. For immune cells, gene variants my alter the way that T and B cells are selected in primary (1°) lymphatic tissues during central tolerance, or they may alter several key events that occur during antigen-specific priming and effector differentiation in the peripheral (2°) lymphatics. Immune destruction of β cells requires homing of innate and adaptive effector populations into the pancreatic islets, so alterations to endothelial function could affect disease at this late stage. Isogenic cellular modeling can be applied to complex multifactorial diseases to facilitate a more complete understanding of which genes are expressed in any given tissue/cell type and at which developmental stage they may exert their influence on disease progression.

The combined genetic and environmental factors that result in type 1 diabetes (T1D) are reflected in the heterogeneous clinical presentations of the disease (1). This autoimmune process results from a complex cross-talk between cells of the innate and adaptive arms of the immune system and the target β cells within the islet microenvironment (Figure 1) (2). The era of genome-wide association studies (GWAS) has heralded discovery of approximately 57 independent loci conferring some component to the overall genetic risk for the development of T1D (3). This vast discovery effort has reinforced prior notions of an autoimmune basis for disease development and also has shed new light on the etiology of T1D, including support for cellular stress within β cells contributing to their demise (4). Despite these advances, there remain numerous questions regarding the mechanisms by which causal gene variants, both individually and in concert, impact immune checkpoints and β cell responses throughout the natural history of the disease. Thus, there remains a critical need in the field to address some fundamental questions regarding the single-nucleotide polymorphisms (SNPs) identified by GWAS including (1) What are the causative variants within any given tag SNP locus? (2) In what cell type(s) and developmental stage(s) are the candidate genes actively expressed? (3) What environmental stimuli modify candidate gene expression or activity? And ultimately, (4) what variants and/or pathways are amenable to therapeutic interventions?

A number of large-scale mechanistic studies to discern the impact of specific genotypes on resulting phenotypes are underway from population-based studies (5). These investigations often utilize clinical material derived from bioresource banks (i.e., genotyped clinical samples capable of recall or recovery from cryopreservation) (6). While promising results have emerged, the number of well-characterized genotype:phenotype interactions remains limited to a small fraction of the putatively identified risk loci. The paucity of functional studies validating causative SNPs can be attributed to a number of challenges including the need to acquire sufficient clinical blood volumes for functional testing, limited access to biological replicates to account for human heterogeneity (particularly with low minor allele frequency variants), and the clear potential for epistatic genetic influences. In sum, these confounding factors constitute a considerable discovery bottleneck limiting human studies by the larger research community.

Immunodeficient mouse models, so-called “humanized” mice, capable of being engrafted with primary human lymphocytes or hematopoietic stem cells (HSCs) have been proposed as a means to fill the translational gap between *in vitro* human studies and clinical trials. These rodent models display full organism level complexity yet can still be manipulated experimentally (7). Despite the powerful tool humanized mice provide when used appropriately, they still present significant constraints as a model system. Mice hold notable differences when compared to human biology, particularly when considering host immune responses in the context of TLR ligands, responses to cytokines and growth factors, and cellular trafficking (8). These factors present challenges in modeling autoimmune T1D in xenogeneic systems, where there are essential homology requirements for full effector function. These requirements include the need for lymphocyte trafficking from circulation to secondary lymphoid organs, auto-antigen priming and activation, and eventual extravasation to target β cells within islets (9). The emergence of induced pluripotent stem cell (iPSC) technologies offers an attractive alternative to humanized mice that allows the interrogation of underlying genetic defects using a vast array of relevant biological tissues and cell types avoiding both allo- and xenogeneic responses.

Isogenic cellular systems constitute a powerful experimental platform for conducting precision gene editing to create a “disease-in-a-dish” model to interrogate multifactorial diseases such as T1D. This methodology provides an opportunity to understand specific molecular mechanisms and pathways in humans to thereby derive rational therapeutics using a precision medicine approach. In this review, we describe some of the emerging technologies for generating and manipulating iPSC-derived cells and tissues to interrogate causative genes and pathways in T1D.

## Isogenic Cellular Systems

**Figure 2:**
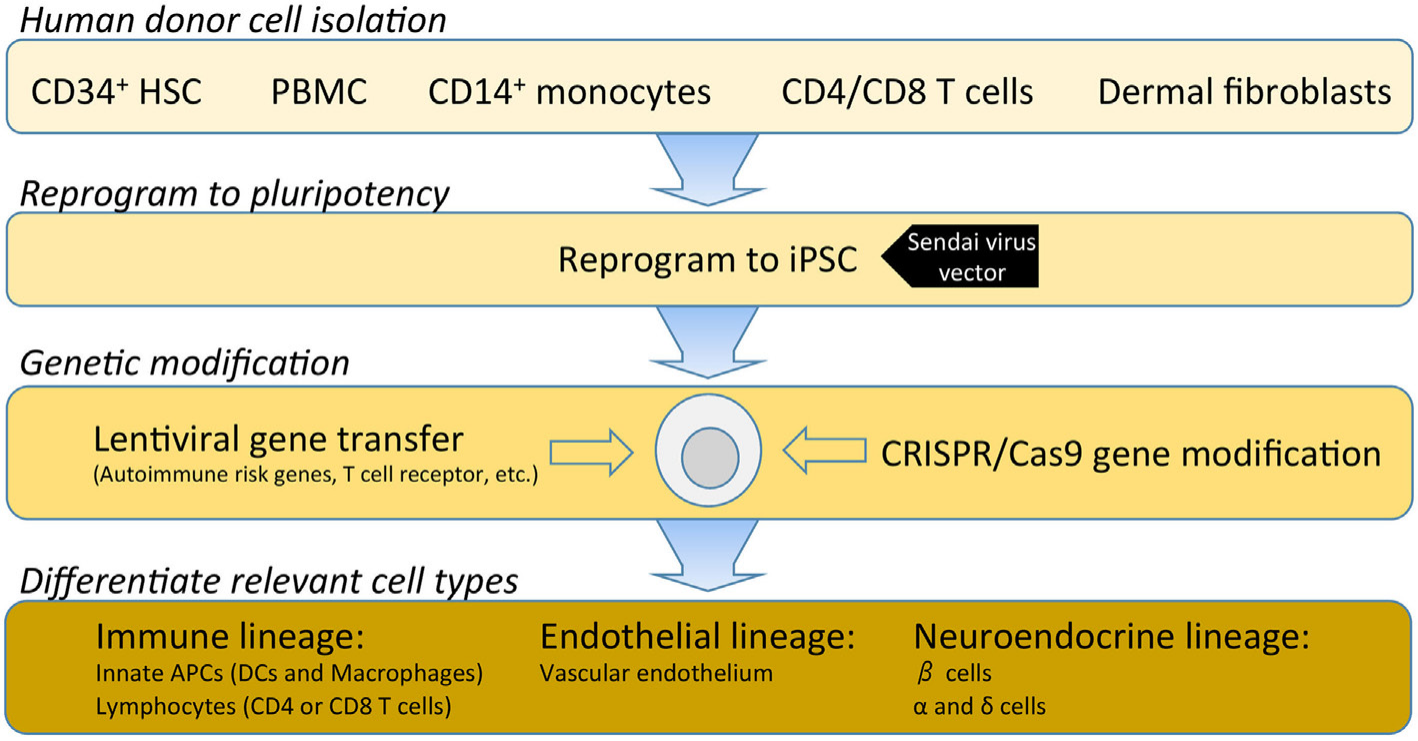
A hypothetical outline for establishing an isogenic disease-in-a-dish workflow. Induced pluripotent stem cell (iPSC) stable cell lines can be generated from several different somatic cell types depending on specimen availability. Traditionally, dermal fibroblasts from skin biopsy were utilized; however, this is being replaced by less invasive samples such as freshly isolated or cryopreserved peripheral blood mononuclear cells (PBMCs). PBMCs can be enriched for various populations such as CD34^+^ hematopoietic stem cells, CD14^+^ monocytes, T cells, or reprogrammed as a bulk population. Where a pre-re-arranged T-cell receptor (TCR) is desired, antigen-specific CD4 or CD8 T cells can be used so that iPSC-derived T cells will clonally express the desired TCR with a naive T-cell phenotype. Several commercial platforms for iPSC reprogramming are currently available. Non-integrating Sendai virus vectors provide a safe and efficient means for iPSC reprogramming of human primary cells. Following reprogramming into iPSC, gene modification enables researchers to investigate disease-associated risk variants and/or over-express or knockdown genes to modulate pathways. Once gene modifications are confirmed, validated protocols for differentiation of immune, endothelial or neuroendocrine lineages are utilized to interrogate the specific effects of each gene variant in several disease-relevant cell types.

Investigations into the etiopathogenesis of T1D have historically been dominated by studies of peripheral blood. Over the last decade, the Network for Pancreatic Organ donors with Diabetes (nPOD) program has provided essential access to the pancreas and lymphoid tissues from donors with T1D. Emerging studies from this program have already challenged many of the preconceived notions of the disease. Of note, nPOD tissues have highlighted disease heterogeneity across T1D donors and remarkable variability even at the level of adjacent islets within a single T1D donor (10–14). For example, early histological observations from nPOD led Dr. George Eisenbarth to refer to T1D as “vitiligo of the pancreas,” in reference to intact insulin-containing islets being observed in close proximity to pseudo-atrophic islets completely devoid of insulin (15). Despite the transformative resource that nPOD provides, donor and programmatic limitations necessitate systematic prioritization of access to tissues. Hence, there is a paramount need within the field to derive cell types from renewable human cellular sources. The capacity for pluripotent and renewable cells to undergo reprogramming to generate immune subsets, endothelial cells, and neuroendocrine lineages will facilitate the modeling of cellular interactions involved in T1D disease pathogenesis (Figure 2).

## Genetic Susceptibility in T1D

**Figure 3:**
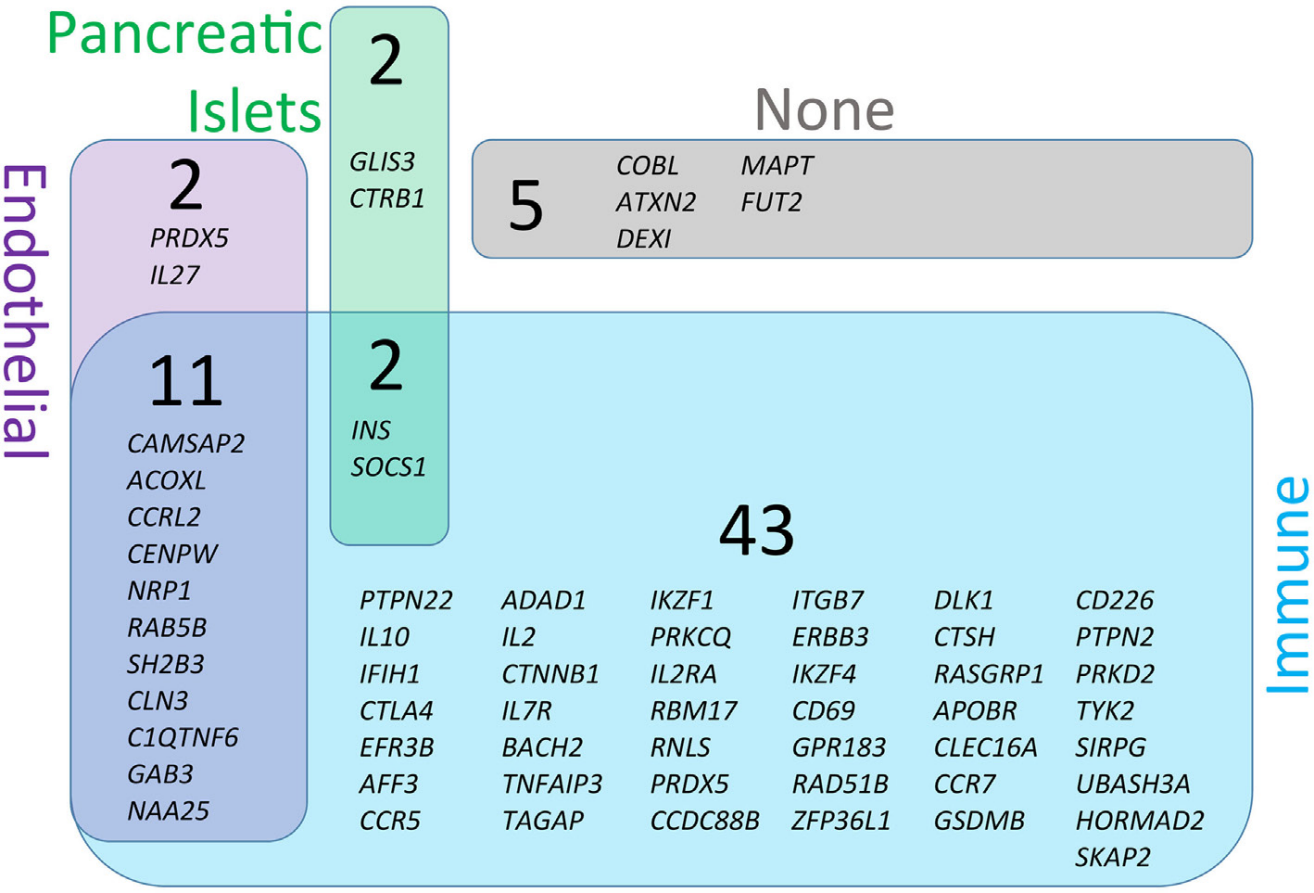
Genetic susceptibility risk variants identified in type 1 diabetes (T1D). The current list of gene regions that have been associated with T1D through genome-wide association studies were collected from the ImmunoBase resource (www.immunobase.org). Individual single-nucleotide polymorphisms (SNPs) corresponding to candidate genes were identified from the ImmunoBase T1D data set. In addition, every SNP tag was queried on the University of California Santa Cruz Genome Browser (GRCh38; genome.ucsc.edu) to identify additional genes in each SNP-tagged region. The complete list of regions and genes are compiled in Table 1. Each SNP-associated gene was queried on the GeneVestigator database to identify the top 10 tissues with highest expression of each gene (genevisible.com/search). For each gene it was determined if high-expressing tissues included any one or combination of relevant tissues: immune (blue), endothelial (purple), or pancreatic islets (green). The size of regions in Venn diagram represents the relative abundance of genes expressed in each tissue type. Five genes were not highly expressed by any of the relevant tissue types (indicated as none, gray).

The autoimmune destruction of insulin-producing pancreatic β cells in T1D shares complex etiology with a collection of organ-specific disorders (i.e., juvenile idiopathic arthritis, alopecia areata, rheumatoid arthritis, and celiac disease, among others) (3). Though each of these diseases demonstrates unique immunopathologic mechanisms, they all share two common features: specifically, inheritance with a significant genetic contribution coming from the human leukocyte antigen (HLA) region of chromosome 6 and additional genetic risk conferred by loci dispersed throughout the genome (Table 1). While no single risk haplotype accurately predicts whether or not a person will develop T1D (or another autoimmune disease), there is clear genetic evidence that T1D is primarily an inherited disease with an autoimmune pathogenesis (Figure 3) and with additional poorly defined environmental contributions. Discordant incidence of T1D in monozygotic twins is often cited as evidence for a greater environmental role in T1D (16, 17); however, the early studies likely underestimated the concordance rates. It is now better understood that childhood-onset T1D and latent autoimmune diabetes of the adult (LADA) share overlapping genetic risk (18). Thus, long-term monitoring is essential to capture the total genetic risk for disease development. For example, one study of monozygotic twins found that by the age of 60 years, there was greater than 65% concordance for T1D—i.e., when one twin is afflicted, it is more likely that the other twin will eventually develop the disease (19). In the same study, concordance of autoantibody positivity in the non-diabetic twin was nearly 80%, again supporting the notion of genetic risk controlling the loss of immune tolerance to β cell antigens.

**Table 1:** Genetic variants associated with type 1 diabetes and other common autoimmune diseases.

Chromosome	Marker	Gene	Feature	Coding variant	Amino acid variation	Additional notes	Region	Other associated diseases
1	rs2476601	*PTPN22*	Exon	Y	R620W		1p13.2	ATD/CRO/JIA/RA/SLE/AA/VIT
	rs6679677		3′ region—intergenic	N				
	rs6691977	*CAMSAP2*	Intron	N			1q32.1	
	rs3024505	*IL10*	3′ region—intergenic	N			1q32.1	CRO/SLE/UC/IBD
	rs3024493		Intron	N				

2	rs35667974	*IFIH1*	Exon	Y	I923V		2q24.2	PSO/SLE/UC/IBD/VIT
	rs2111485		3′ region—intergenic	N				
	rs1990760		Exon	Y	A946T			
	rs11571316	*CTLA4*	5′ region—intergenic	N			2q33.2	ATD/CEL/RA
	rs3087243		3′ region—intergenic	N				
	rs4849135	*ACOXL*	Intron	N			2q13	
	rs478222	*EFR3B*	Intron	N			2p23.3	
	rs9653442	*AFF3*	5′ region—intergenic	N			2q11.2	RA

3	rs113010081	*CCR5* and	3′ region—intergenic	N			3p21.31	CEL/UC
		*CCRL2*						

4	rs2611215	*LINC01179*	5′ region—intergenic	N			4q32.3	
	rs75793288	*CTNNB1*	Intron	N		5′ of ADAD1 and 3′ of IL2	4q27	CEL/CRO/UC
	rs6827756		Intron	N		5′ of ADAD1 and 3′ of IL2		
	rs4505848		Intron	N		5′ of ADAD1 and 3′ of IL2		
	rs17388568	*ADAD1*	Intron	N		3′ of IL2		
	rs10517086	*No gene*	Intergenic—H3K27Ac rich	N			4p15.2	

5	rs11954020	*IL7R*	3′ region—intergenic	N			5p13.2	

6	rs9388489	*CENPW*	Intron	N			6q22.32	
	rs1538171		Intron	N				
	rs9375435		Intron	N				
	rs597325	*BACH2*	Intron	N			6q15	ATD/MS/RA
	rs11755527		Intron	N				
	rs72928038		Intron	N				
	rs924043	*No gene*	Intergenic	N			6q27	
	rs6920220	*TNFAIP3*	5′ region—intergenic	N			6q23.3	RA/SLE/UC/IBD
	rs1738074	*TAGAP*	Exon	N	SYN		6q25.3	CEL/MS

7	rs7804356	*SKAP2*	Intron	N			7p15.2	
	rs4948088	*COBL*	3′ region—intergenic	N			7p12.1	
	rs62447205	*IKZF1*	Intron	N			7p12.2	

9	rs10758593	*GLIS3*	Intron	N			9p24.2	
	rs7020673		Intron	N				
	rs6476839		Intron	N				

10	rs722988	*NRP1*	3′ region—intergenic—H3K27Ac rich	N			10p11.22	
	rs11258747	*PRKCQ*	Exon	N	SYN		10p15.1	
	rs61839660	*IL2RA*	Intron	N			10p15.1	MS/RA
	rs2104286		Intron	N				
	rs12251307	*IL2RA* and *RBM17*	5′ of RMB17 and 3′ of IL2RA	N				
	rs41295121		5′ of RMB17 and 3′ of IL2RA	N				
	rs7090530		5′ of RMB17 and 3′ of IL2RA	N				
	rs10795791		5′ of RMB17 and 3′ of IL2RA	N				
	rs12416116	*RNLS*	Intron	N			10q23.31	
	rs10509540		3′ region—intergenic	N				

11	rs72853903	*INS*	5′ region—intergenic—H3K27Ac rich	N			11p15.5	
	rs689		Intron	N				
	rs7111341		5′ region—intergenic	N				
	rs7928968		3′ region—intergenic	N				
	rs694739	*BAD*	5′ region—intergenic	N		5′ of CCDC88B and 3′ of PRDX5	11q13.1	CRO/MS/AA

12	rs11170466	*ITGB7*	Intron	N			12q13.13	
	rs11171739	*ERBB3*	5′ region—intergenic	N			12q13.2	AA
	rs11171710	*RAB5B*	Intron	N		5′ of IKZF4		
	rs705705	*IKZF4*	3′ region—intergenic	N				
	rs705704		3′ region—intergenic	N				
	rs2292239	*ERBB3*	Intron	N				
	rs3184504	*SH2B3*	Exon	Y	R262W		12q24.13	CEL/CRO/JIA/PBC/RA/AA/PSC/VIT
	rs653178	*ATXN2*	Intron	N				
	rs17696736	*NAA25*	Intron	N				
	rs10492166	*CD69*	3′ region—intergenic	N			12p13.31	
	rs4763879		Intron	N				

13	rs9585056	*GPR183*	5′ region—intergenic—H3K27Ac rich	N			13q32.3	

14	rs4900384	*LINC01550*	5′ region—intergenic	N			14q32.2	
	rs1456988		5′ region—intergenic	N				
	rs911263	*RAD51B*	Intron	N			14q24.1	PBC
	rs1465788	*ZFP36L1*	5′ region—intergenic—H3K27Ac rich	N			14q24.1	
	rs56994090	*DLK1*	3′ region—intergenic	N		Intron of MEG3	14q32.2	
	rs941576		3′ region—intergenic	N		Intron of MEG3		

15	rs12148472	*CTSH*	Intron—splice site	N			15q25.1	CEL/NAR
	rs3825932		Intron	N				
	rs34593439		Intron	N				
	rs12908309	*RASGRP1*	5′ region—intergenic	N			15q14	CRO
	rs72727394		Intron	N				

16	rs4788084	*IL27*	5′ region—intergenic	N			16p11.2	AS/CRO/IBD
	rs9924471		5′ region—intergenic	N		Intron of *SGF29*		
	rs151234	*CLN3*	Intron	N		5′ of *APOBR* and 3′ of *IL27*—K3K27Ac rich		
	rs12708716	*CLEC16A*	Intron	N			16p13.13	MS/PBC
	rs12927355		Intron	N				
	rs193778	*SOCS1*	5′ region—intergenic—H3K27Ac rich	N		3′ of *CLEC16A*, Intron of *RMI2*		
	rs8056814	*CTRB1*	5′ region—intergenic—H3K27Ac rich	N			16q23.1	
	rs7202877		5′ region—intergenic—H3K27Ac rich	N				

17	rs1052553	*MAPT*	Exon	N	SYN		17q21.31	
	rs7221109	*CCR7*	5′ region—intergenic—H3K27Ac rich	N			17q21.2	
	rs2290400	*GSDMB*	Intron	N			17q12	CRO/UC/IBD
	rs12453507		3′ region—intergenic	N				

18	rs763361	*CD226*	Exon	Y	G307S		18q22.2	MS
	rs1615504		3′ region—intergenic	N				
	rs2542151	*PTPN2*	3′ region—intergenic	N			18p11.21	CEL/CRO/UC/IBD
	rs1893217		Intron	N				

19	rs602662	*FUT2*	Exon	Y	G258S		19q13.33	CRO/IBD
	rs516246		Intron	N				
	rs402072	*PRKD2*	Intron	N			19q13.32	
	rs425105		Intron	N				
	rs12720356	*TYK2*	Exon	Y	I684S		19p13.2	CRO/JIA/MS/PBC/PSO/RA/IBD
	rs34536443		Exon	Y	P1104A			

20	rs2281808	*SIRPG*	Intron	N			20p13	
	rs6043409		Exon	Y	V263A			

21	rs11203202	*UBASH3A*	Intron	N			21q22.3	RA/VIT
	rs11203203		Intron	N				

22	rs4820830	*HORMAD2*	Intron	N			22q12.2	
	rs5753037		3′ region—intergenic	N				
	rs229533	*C1QTNF6*	5′ region—intergenic	N		3′ of *RAC2*	22q12.3	

X	rs2664170	*GAB3*	Intron	N			Xq28	

*Genes and markers were derived from immunobase.org (3, 20–26). The genes indicated in *blue text* were imputed from information derived from the University of California Santa Cruz genome browser (genome.ucsc.edu). Amino acid variations (*red text*) were identified for single nucleotide polymorphism (SNP) variants by downloading the spliced coding sequences from genome browser and translating in SnapGene software. SYN (*green text*) indicates synonymous variation in an exon. Genomic region and disease information displayed were derived from Immunobase*.

*ATD, autoimmune thyroid disease; CRO, Crohn’s disease; JIA, juvenile idiopathic arthritis; RA, rheumatoid arthritis; SLE, systemic lupus erythematous; AA, alopecia areata; VIT, vitiligo; UC, ulcerative colitis; IBD, inflammatory bowel disease; PSO, psoriasis; CEL, celiac disease; MS, multiple sclerosis; PBC, primary biliary cirrhosis; PSC, primary sclerosing cholangitis; NAR, non-allergic rhinitis; AS, ankylosing spondylitis*.

The lack of complete concordance may indicate an additional role for epigenetic and/or stochastic influences due to antigenic receptor gene recombination events. In addition, epidemiological studies support a role for environmental factor(s) influencing disease progression. A number of large consortium studies have been conducted or are currently underway around the world (e.g., TrialNet, TEDDY, DAISY, BABYDIAB, and Pre-Point) to monitor disease progression and potentially, intervene in those identified as being at high-risk for disease development (27–31). From these studies, environmental influences have been reported to affect disease incidence or rate of progression, including enteroviral triggers, lack of protective exposures, and the influence of various components of Westernized diets. Many of these modifying factors impact pathways with associated genetic risk variants (e.g., *Tyk2* and *IFIH1* in response to viral infections), further supporting their potential importance (32). Thus, T1D is principally a genetic disease with environmental exposures influencing progression. These combined influences support the notion of a complex multifactorial disease, yet ultimately beg the question: Why do we not better understand the etiology and pathogenesis of human T1D? Even though the human genome is complex, it is still a finite collection of variables. In principle, utilization of “big data” approaches involving GWAS, biomarker studies, and expression profiling, when paired with robust computational capabilities, should be able to reveal a clear molecular signature, and from this signature, we should be able to progress through reductionist approaches to reveal pathways of disease.

This theoretical solution to the problem of complex autoimmune diseases is hindered by a number of fundamental challenges. Foremost, heretofore there have been no experimental systems available to study individual risk variants in human subjects. For T1D, where approximately 57 different genetic regions confer some portion of genetic risk (immunobase.org, July 2017) (3, 20–26), it is not possible to study one gene at a time without incurring significant epistatic effects from other risk genes. The likelihood of finding two individuals differing at *only* one risk gene (i.e., one person with the protective allele and one person with the risk allele) while having *identical* variants at the remaining 56 risk regions is infinitesimally small. A more practical approach would be to reduce the number of genetic loci being studied to include only those with the largest odds ratios (ORs). Even here, the problem is magnified by the fact that some of the most highly associated risk genes beyond the HLA [e.g., protein tyrosine phosphatase, non-receptor type 22 (*PTPN22*)] have a low minor allele frequency, even among T1D subjects. For North American and European T1D subjects, the frequency of individuals with homozygosity for the risk variant of *PTPN22* (1858T at rs2476601) ranges from 0.6 to 3.7% (33). Moreover, genes associated with T1D risk encode proteins that cluster within biological processes and/or pathways, posing a considerable challenge when analyzing the impact of a given risk variant.

Currently, 57 genomic regions that are defined by 104 SNPs [some SNPs identify the same linkage disequilibrium block] are significantly associated with T1D according to immunobase.org. The set of 64 T1D candidate gene variants from 57 SNP-tagged regions listed in Table 1 was analyzed using the Protein ANalysis THrough Evolutionary Relationships gene ontology tool (pantherdb.org) (34, 35). Not surprisingly, pathway analysis revealed a significant enrichment for genes involved in immune processes (*P* = 9.9E−11), where 26 of the 64 candidate genes contribute to immune function. The immune system is highly dynamic and integrates signals from antigenic receptors, adhesion molecules/integrins, costimulatory molecules, and cytokine/chemokine receptors. These events in turn lead to signal transduction events that are also significantly enriched as a defined pathway. Based on our analysis, 32 of the 64 T1D candidate genes are implicated in cellular signaling (*P* = 9.25E−03) (Data File S1 in Supplementary Material). Considering the role of cross-talk between signaling pathways, it is evident that heterogeneous genetic risk will result in complex downstream effects on cell signaling and functions.

As a specific example of immune signaling pathway cross-talk, we consider one gene that encodes a protein with known effects on cytokine receptor signaling. *SH2B3* encodes a protein phosphatase Lnk that regulates Janus kinase/signal transducers and activators of transcription (JAK/STAT) signaling. The risk variant of *SH2B3* (T at rs3184504) encodes a modified Lnk protein where arginine at amino acid 262 is replaced by tryptophan (R262W). Lnk is a regulator of Jak2 signaling in myeloid cells (36–38), and the T1D risk SNP for *SH2B3*/Lnk is associated with altered expression of key elements of IFNγ signaling including signal transducer and activator of transcription 1 (STAT1) (39). Furthermore, the target of Lnk, Jak2, is a cytosolic protein that transduces signals from a variety of cytokine receptors including IL-6, IL-13, G-CSF, IL-12, IL-23, granulocyte-macrophage colony-stimulating factor (GM-CSF), EPO, IL-3, and IL-5 (40, 41). Thus, the specific effect(s) of Lnk^R262W^ upon immune cell function are difficult to predict. Adding to this inherent complexity, additional T1D risk genes/proteins are likely to co-regulate the same pathways as Lnk. For example, at least three T1D candidate genes, *Tyk2, SOCS1*, and *IL10*, encode proteins with known roles in modulating JAK/STAT signaling. The interplay of different alleles of each protein will likely modify the effect of Lnk. This example highlights the need for an experimental system that mitigates the epistatic effects of related genes/proteins so that observed phenotypes are attributed to the gene of interest alone.

In addition to the number of variants and overlapping pathways noted above, there are additional layers of complexity at the cellular level. Specifically, it is poorly characterized how a given risk variant may impact function within various innate or adaptive immune subsets. For example, a gene that regulates JAK/STAT signaling in antigen-presenting cells (APCs) such as dendritic cells (DCs) may have an entirely different biological effect in lymphocytes. Moreover, the impact of a gene variant may be combinatorial to multiple cell types that conspire to drive autoimmunity. Furthermore, some genes may affect the β cells themselves, endothelial cells, or other cells such as neurons (42).

The central pathophysiological mechanism of T1D entails at least three major tissue types—immune, endothelial, and pancreatic (Figure 1). To better understand which cells are likely to be affected by each T1D candidate gene, we analyzed all genes from Table 1 for cell/tissue expression profiles using the online GeneVisible tool (genevisible.com) (43) that queries tens of thousands of curated human gene expression experiments. As seen in Figure 3, the majority of T1D candidate genes are expressed most highly in immune cells, but a small number of genes are preferentially expressed in endothelial or pancreatic cell lineages. Notably, 13 genes are highly expressed in multiple lineages. Isolating the effect of candidate genes in relevant cell types should be a goal for the isogenic cellular experimental system described herein.

A number of T1D-associated SNPs encode missense mutations within gene exons, presumably altering protein stability, interactions, or function (Table 1); here, the path to dissect the impact of variants on biological processes is straightforward. However, the vast majority of risk loci reside in non-coding regions of the genome and careful studies must be undertaken to first dissect the causative variant(s) from each tag SNP locus and then determine whether any given SNP exerts its impact in a *cis* or *trans* manner to alter gene expression (5). One such study by Ram et al. recently applied a systems genetics approach to dissect the impact of putative risk SNPs on gene expression in purified and activated cell lines. The authors mapped *cis*-acting expression quantitative trait loci (eQTL) and found 24 non-HLA loci that significantly affected the expression of 31 transcripts in at least one cell type from Epstein–Barr virus-transformed B cells and CD4^+^ or CD8^+^ T cells (44). They went on to describe an additional 25 *trans-*acting loci that impacted 38 transcripts. Of note, many of the SNPs associated with risk are located within promoter or enhancer regions of their candidate gene (3). These studies provide a framework from which additional mechanistic studies can now be conducted in isogenic cellular systems.

**Figure 4:**
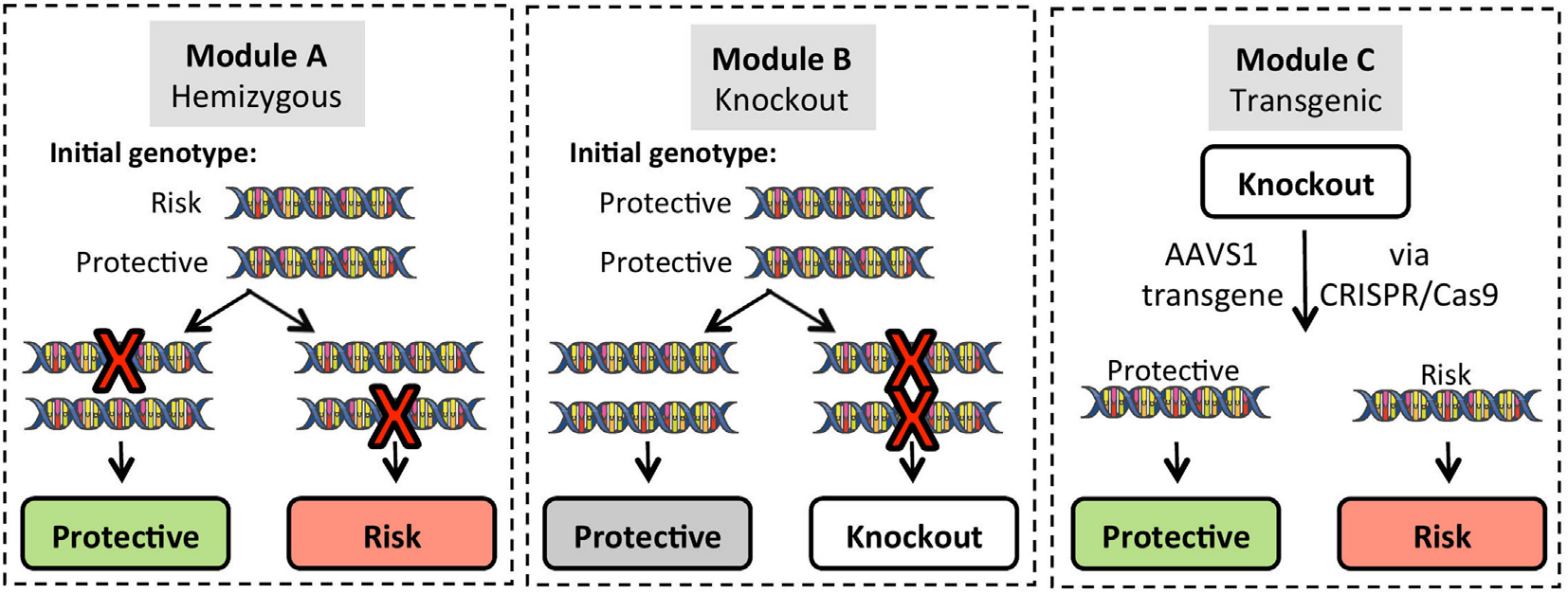
Gene modification strategies for use in induced pluripotent stem cell (iPSC) lines. Three basic strategies can be employed for gene editing. To create single allele homozygous expression (hemizygous) lines, *Module A* targets a single allele of the gene of interest (GOI) in iPSC lines that are heterozygous for the risk variant. Targeting efficiency for hemizygous clones is approximately 20% of green fluorescent protein positive puromycin resistant clones, and allele targeting is random so that either the protective or risk allele can be modified. *Module B* generates complete knockout of the GOI on a background of homozygous protective alleles. *Module C* utilizes GOI-knockout lines to re-express either the protective or risk variant of the GOI using a CRISPR/Cas9 platform that targets integration into the adeno-associated virus integration site 1 (AAVS1) on chromosome 19.

To begin to address these challenges, the research community needs robust platforms to study the effects of individual risk alleles in various cell types under controlled conditions. With the advent of iPSC technologies and genome-editing tools, this once theoretical approach now provides an efficient method to analyze disease mechanisms and identify causal gene variants (Figure 4). By creating a disease-in-a-dish experimental platform, we and others have started to dissect the individual contributions of T1D risk genes in specific cell types. Harnessing this information will allow researchers to derive rational therapeutics targeting checkpoints in key pathways.

## Defective Immune Tolerance in T1D

Autoimmune diseases, including T1D, result from a breakdown in the pathways that maintain a state of immune homeostasis, commonly referred to as immune tolerance (1). The mechanisms controlling this process involve both central and peripheral tolerance mechanisms (e.g., thymic selection and immune checkpoints, respectively). Effective immunity requires the capacity to respond to a vast array of antigens from pathogens, all while functionally limiting host responses to self-tissues and commensal microorganisms. In health, the adaptive immune system consisting of T and B lymphocytes is edited to eliminate portions of the repertoire that acquire somatically re-arranged receptors with high binding affinity for self-antigens through clonal deletion. For T cells, this process is carried out in the thymus under the control of the autoimmune regulator (AIRE) (45). Medullary thymic epithelial cells (mTECs) expressing AIRE are thought to coordinate the expression of a select number of tissue-specific self-antigens (TSAs). These TSAs, when expressed by mTEC work in concert with APCs to eliminate high-affinity autoreactive T-cell receptors (TCRs) through negative selection. The thymus is also the site for the emergence of thymic CD4^+^ regulatory T cells (tTregs), a population of cells that express the master Treg-transcription factor FOXP3 (46). These tTregs seed the periphery, playing a key role in reinforcing immune tolerance. Rare monogenic mutations in *AIRE* and *FOXP3* result in profound autoimmune conditions, referred to as autoimmune polyglandular syndrome-1 and immunodysregulation polyendocrinopathy enteropathy X-linked syndrome (IPEX), respectively (47). T1D is a common clinical manifestation of patients presenting with these severe mutations, supporting the essential role for these transcription factors in regulating immune tolerance to β cells. To date, studies of thymic T-cell selection have largely been restricted to animal models. The development of isogenic cellular systems provides a unique opportunity to investigate molecular pathways that shape the human adaptive immune repertoire.

## Isogenic Models for Studying Thymic Selection

As noted earlier, the HLA region constitutes the major genetic risk locus in T1D (48). While this region has been known to confer risk for over four decades, the exact mechanisms by which variants in HLA influence disease pathogenesis remain poorly characterized. In addition to shaping the T-cell repertoire through the processes of positive and negative selection, the thymic developmental niche controls the composition and relative proportion of naive conventional T cells (Tconv) and tTregs that emerge to form the mature CD4^+^ T cell population (49). Little is currently known about how high-risk HLA haplotypes (e.g., HLA-DR3/DR4-DQ8) shape the resulting T-cell repertoire, or for that matter, why the HLA-DRB1*15:01-DQA1*01:02-DQB1*06:02 haplotype is so dominantly protective in Caucasian populations (OR ~0.03) (50). A prevailing theory presented by Eisenbarth and colleagues suggests that the key might lie within the tri-molecular complex of HLA class II molecules presenting peptides of insulin (specifically, the insulin B-chain_9-23_) for recognition by autoreactive TCRs (51). T1D DQ8 risk alleles and I-A^g7^ of the non-obese diabetic (NOD) mouse tend to share non-polar residues in place of Asp at β57 and preferentially bind peptides with acidic side chains in the P9 pocket of the MHC class II binding groove (52). Thus, these molecular interactions within distinct peptide binding pockets may either allow escape of potentially pathogenic autoreactive T-cell clones from the thymus or potentially fail to generate the proper repertoire of protective tTregs capable of maintaining tolerance.

Together with HLA, additional candidate risk genes could also have an impact on thymic T-cell development. Specifically, at least three independent variants within the *INS-IGF2* locus have been associated with risk for T1D (3). This region confers the second highest risk for disease following the HLA locus. Risk associated with the *INS* gene on chromosome 11p15.5 has been most commonly attributed to a variable number tandem repeat locus situated 596 bp 5′ of *INS* (53). Protection from the class III allele has been attributed to a markedly higher level of insulin being expressed within the thymus (54). Insulin has been proposed as a primary or triggering auto-antigen in the NOD mouse model (55) and more recently in human T1D (56). Notably, T cells reactive to both native and hybrid insulin peptides, insulin conjugated with other β cell antigens, were discovered within the islets of subjects with T1D (56–58). When considered in addition to the dominance of genetic risk conferred by the HLA, these significant observations lend additional support to the dominance of insulin epitopes as a primary auto-antigen in disease pathogenesis.

These reports highlight the need for mechanistic studies to ascertain how susceptibility alleles impact the process of thymic selection. Through the creation of isogenic systems involving human bone marrow progenitors, thymic organoids, mTECs, and APCs, novel avenues can now be explored to investigate genetic control of the human adaptive T-cell repertoire. Key polymorphisms may be altered by gene editing and genes and/or pathways may be “switched” on or off in a temporal fashion by the addition of chemical enhancers or repressors in either T-cell precursors or thymic stroma. Not only will this provide key insight into pathogenic versus regulatory receptors but could also potentially provide an opportunity for the *ex vivo* education of T cells in isogenic thymic organoids for auto- and/or allo-tolerance induction strategies following β cells regenerative or replacement therapies in T1D.

## Modeling Antigen-Specific T-Cell Responses

Type 1 diabetes is most often described as a T-cell-mediated organ-specific autoimmune disease. This notion emanates from seminal experiments including the strong linkage to HLA, early animal model adoptive transfer experiments (59, 60), and the presence of autoreactive memory T cells within the insulitic lesion of organ donors with T1D (12, 14). Studies have been conducted to investigate and monitor autoreactive T cells in peripheral blood mononuclear cell (PBMC) of T1D subjects. To date, none of the commonly employed techniques have approached the sensitivity/specificity and level of standardization observed for autoantibody assays validated by the diabetes antibody standardization program now known as the islet autoantibody standardization program (61–66). We would speculate that the major reason(s) for this inability to identify robust T-cell biomarkers results from both technical limitations of the current assays, along with the inherent biology of T cells. Our data profiling the TCR repertoire in T1D nPOD organ donors demonstrated only modest overlap in high frequency clonotypes between the pancreatic lymph nodes and spleen (as a surrogate of PBMC) (67). This was particularly striking for CD4^+^ T cells (mean ± SD; 9.2 ± 7.0% of clones shared), with CD8^+^ T cells demonstrating significantly more TCR-β complementarity determining region 3 amino acid sequence overlap among different tissues (36 ± 21%).

Studies to quantify antigen-specific T cells with ELISpot or MHC-multimer reagents have demonstrated the rare nature of these cells in PBMC (in the range of 1:50,000–1:1,000,000) (68). This presents a number of challenges when trying to identify key auto-antigen targets and peptides important during the natural history of disease. To address this particular limitation, we have co-opted an approach pioneered in the cancer immunotherapy field to generate large numbers of tumor-antigen-specific T cells. Specifically, we have generated lentiviral contructs that express full TCR-α and β chains in multi-cistronic expression cassettes. This technique is effective for redirecting the specificity of primary human Tconv and Tregs as well as CD8^+^ T cells (69). Recent advances in gene editing and receptor engineering have advanced this field to create programmable circuits for studying T-cell specificity and effector functions (70). Importantly, we have recently employed TCR gene transfer to directly test the cytotoxic activity of glucose-6-phosphatase-reactive CD8^+^ T cells to target and lyse β-Lox5 cells or primary β cells *in vitro* (71). From a therapeutic perspective, our current efforts demonstrate that human Tregs can be redirected to recognize β cell auto-antigens in the context of DR3/DR4-DQ8 and remain highly suppressive *in vitro* to Tconv recognizing a shared peptide or in a bystander fashion (72). The application of novel single cell/clone analysis platforms, when used in concert with isogenic cellular systems, will allow researchers to quickly move from *in silico* TCR-α/β sequence information to unlimited numbers of antigen-specific T cells to expedite auto-antigen discovery and functional studies.

iPSCs can be used for yet another approach to generate a large number of antigen-specific T cells and to further study mechanisms of thymic selection. A small number of groups have successfully differentiated iPSCs into functional T cells. iPSC derived from a single CD8^+^ T-cell clone have been re-differentiated into naive and eventually highly functional CTLs (73). This application has emerged as a particularly potent means to not only bolster the number of antigen-specific T cells but also correct the anergic and senescent phenotype common to tumor-infiltrating T cells in cancer, and while early studies were focused on generation of CD8^+^ CTLs for targeting virus-infected cells (73) or tumors (74), the methods could be adapted to focus on auto-antigen-specific T cells. When iPSC derived from non-T cells (not bearing re-arranged TCR genes) are used for T-cell differentiation, a broad diversity of TCR rearrangement events is possible (75).

Differentiation protocols for iPSC-derived T cells require culture on the murine stromal cells line OP9 expressing the Notch ligand protein DL1 (74). The quality of iPSC-derived T cells has been incrementally improved by altering culture conditions, for example activating CD4/CD8 double positive iPSC-derived thymocytes via CD3 to enhance CTL killer activity (76). Today, detailed protocols are available for the differentiation of antigen-specific CD8^+^ T cells from iPSC (73). To date, advances in single-positive CD4^+^ T cells have not approached the same progress as CTLs, yet efficient protocols to generate CD4^+^ T_H_-cell populations are expected. For example, advances in deriving human thymic epithelial cells from iPSCs (77) could enhance *in vitro* differentiation of CD4^+^ and CD8^+^ T cells by providing the full repertoire of human soluble and membrane-associated growth factors. In addition, iPSC-derived thymic epithelia will enable more precise studies of how disease-associated gene variants impact thymic selection by regulating specific processes such as auto-antigen expression during negative selection. The capacity to grow and differentiate large numbers of isogenic antigen-specific T cells (>10^9^ cells), without the typical constraints of primary human T-cell clones opens up the potential for gene editing and extensive functional studies. Thus, we are nearing the point where isogenic iPSC systems can be used to study human T-cell development at a mechanistic level that was previously only attainable in animal models.

## Modeling Innate Immune Responses

Development of auto-antigen-specific T cells requires more than a failure of thymic negative selection. Naive T cells in the periphery must be primed by professional APCs. DCs are specialized APCs with potent abilities to initiate antigen-specific CD4^+^ and CD8^+^ T-cell responses. To elicit CD4^+^ T cells priming, activation, proliferation, and effector function, DCs must first capture antigens via phagocytosis or micropinocytosis. It can be envisioned that this antigen capture in T1D manifests through DCs phagocytosing dead/dying β cells or exosomes derived from β cells.

Several genes associated with T1D risk are expressed in myeloid lineages including monocytes, macrophages, and DCs, and it is likely that at least some of the immune pathogenesis of T1D arises from the innate end of the immune system. Differences in innate immune function could emanate from dysregulated antiviral or type 1 interferon (T1-IFN) responses, altered co-stimulation, changes in antigen acquisition, or enhanced expression of pro-inflammatory cytokines. As an example, a T1-IFN response signature has been observed preceding T1D onset in high-risk populations (32). The NOD Rip-LCMV mouse model corroborates this finding, where IFN-α is critical for progression of T1D (78). Furthermore, some enteric viral infections have been associated with risk for T1D. In NOD mice, rotavirus infection can accelerate T1D in a T1-IFN-dependent manner (79). In humans, a growing number of studies have reported associations between enterovirus infection and T1D (80–84). Thus, genes that regulate the innate response to viruses including T1-IFN expression or signaling could mediate T1D risk by altering innate immune function.

## T1D Risk Genes That Modulate Antiviral Immunity

PTPN22, commonly associated with modifying receptor signaling in T and B cells, is also reported to alter the way that DCs respond to danger signals such as bacterial lipopolysaccharide by modulating TRAF3 signaling and T1-IFN production (85). In lupus, the risk variant of *PTPN22* tagged by rs2476601, the same variant that is associated with T1D (Table 1), is associated with altered TLR7-induced T1-IFN production (86).

A major counter-regulator of IFN signaling is the regulatory cytokine IL-10. Indeed, IL-10 is so potent for protection of host cells from CTL-mediated killing that many DNA viruses have evolved viral homologs of IL-10 to protect them from antiviral immunity (87). The T1D risk locus defined by the SNPs rs3024504 and rs3024493 includes *IL10* (Table 1). A protective role for IL-10 in murine T1D has been established through transgenic NOD mice that over-express IL-10 or where exogenous administration of recombinant IL-10, plasmid DNA encoding IL-10, or cells expressing IL-10 have been used (88–90). Moreover, *in vitro*, IL-10 protects human islets from the cytotoxic effects of inflammatory cytokines (91).

From the innate arm of the immune system, variant alleles of the T1-IFN receptor downstream signaling protein Tyk2, the cytosolic viral RNA sensor IFIH1 (MDA5), the macrophage lysosomal enzyme cathepsin H, and the phosphatase *SH2B3* are also associated with risk for T1D (Table 1). Collectively, these genes along with *PTPN22, IL10, SOCS1* and potentially others signify a major role for innate immune responses in T1D pathogenesis. Similar to T-cell responses, isogenic systems are critical for understanding how each risk variant affects innate immune function.

## Isogenic Modeling of Innate-Adaptive Immune Interactions

Innate APCs participate in the initiation of immune responses; however, they also play an important role in sustaining an ongoing adaptive immune response. Interaction of APCs with antigen-specific CD4^+^ T cells provides bi-directional signals to both cell types. CD4^+^ T helper type 1 (T_H_1) cells are important enhancers of macrophage function. Secreted cytokines (e.g., IFN-γ) and membrane-associated so-stimulatory molecules [e.g., CD40 ligand (CD40L)] expressed by T_H_1 cells arm macrophages to more effectively kill microbes or infected cells. In T1D pathogenesis, there are essential roles for T_H_1 T cells, IFN-γ, CD40-CD40L, and intra-islet macrophages. Where IFN-γ-secreting T_H_1 cells encounter macrophages in the islets of NOD mice, the macrophages become activated and produce inflammatory cytokines and reactive oxygen species that kill β cells (92).

Most human studies of macrophages and DCs rely on two sources of cells—transformed monocytic leukemia cell lines or peripheral blood monocytes isolated from venipuncture. Some studies utilize alveolar macrophages derived from broncioloaviolar lavage or other specialized macrophages that are collected and studied *ex vivo*; however, sample number and size are limiting. PBMCs, while plentiful in number, easy to differentiation into macrophages or DCs, and available from large cohorts due to the low risk of venipuncture, are not ideal for all genetotype:phenotype studies where as discussed above, isogenic systems are key. This is further complicated in monocytes, macrophages and DCs because they are non-dividing cells in culture and generally difficult to modify genetically. iPSCs offer a solution to both problems because they are relatively simple to modify by lentiviral gene delivery or CRISPR/Cas9 and they are effectively immortal in culture. Differentiation of monocytes from iPSC offers the opportunity to study individual T1D risk genes in macrophages and DCs with unprecedented clarity. Protocols for differentiation of iPSC-derived monocytes vary widely from a simple two-cytokine mix of IL-3 and macrophage colony-stimulating factor (MCSF) (93) to a complex mix of cytokines and growth factors (94). Both protocols yield monocytes that can be differentiated into macrophages or DCs using standard conditions (MCSF for macrophages; GM-CSF + IL-4 for DCs), and the differentiated cells retain functional properties of peripheral blood monocyte-derived cells. Thus, isogenic systems now allow researchers to study the effects of a gene variant in either adaptive or innate immune cells alone, but more importantly, we can now determine how T1D risk variants impact innate/adaptive immune interactions, which are more representative of *in vivo* disease etiology.

## The β Cell and Islet Microenvironment

While the immune system is thought to serve as the primary pathogenic mediator of T1D, there are events leading up to that cytotoxic cell–cell interaction that must occur to facilitate autoreactive T-cell destruction of β cells. Specifically, autoreactive CD8^+^ T cells must home from the bloodstream and tether to inflamed endothelium creating firm adhesion contacts, extravasate through the endothelial membrane into the extracellular matrix (ECM), and eventually survey the microenvironment for their cognate antigens presented by HLA class I hyperexpressing islets (95). To completely model the events driving immune destruction of β cells *in vitro*¸ culture systems are needed where both β cells and endothelium can be derived. Extensive research has focused on the differentiation of functional, glucose-responsive, insulin-secreting β cells from human embryonic stem cells (hES) (96–98) as well as iPSC (99–103). Established protocols rely upon multistage culture of pluripotent cells to derive definitive endoderm followed by progressive differentiation of pancreatic endoderm. Often the β cells (or β-like cells) are transplanted to immunodeficient mice where further maturation and functional development continue *in vivo* (104–106). More recently, methods have been developed to convert human fibroblasts into β-like cells by compressing the differentiation protocol so that iPSC reprogramming and differentiation of endoderm occur simultaneously (107). Many of these efforts are being carried out with the eventual goal of replacing β cell mass in T1D patients or utilizing xenotransplantation into humanized mice to model T1D pathogenesis. An alternative is to use β-like cells and immune cells from syngeneic iPSC to model immune destruction of β cells *in vitro*. This process could include endothelial layers (108–110), or ECM barriers that mimic key structures involved in immune homing *in vivo*. The advantage of this specific approach would include the ability to test novel strategies for blocking cellular adhesion, chemotaxis to inflammatory chemokines (e.g., IP-10), as well as potentially blocking degradation of the ECM needed for T-cell migration into the islet microenvironment.

## Isogenic Cellular Systems: A Tool for Expediting Translational Therapies

The emerging fields of iPSC and isogenic cellular systems, when coupled with genome-editing technologies, hold great potential for elucidating causative genes in complex disorders such as T1D. With at least 57 independent genetic variants contributing to overall risk, the need for experimental platforms to expedite validation of causal variants is paramount to the field of functional genomics. Before starting an iPSC project, a few considerations must be made: (1) What will be the source material for iPSC reprogramming (i.e., risk gene profile)? (2) Which of several available iPSC reprogramming methods will be utilized? and (3) What differentiation protocols are available for the cells of interest?

**Figure 5:**
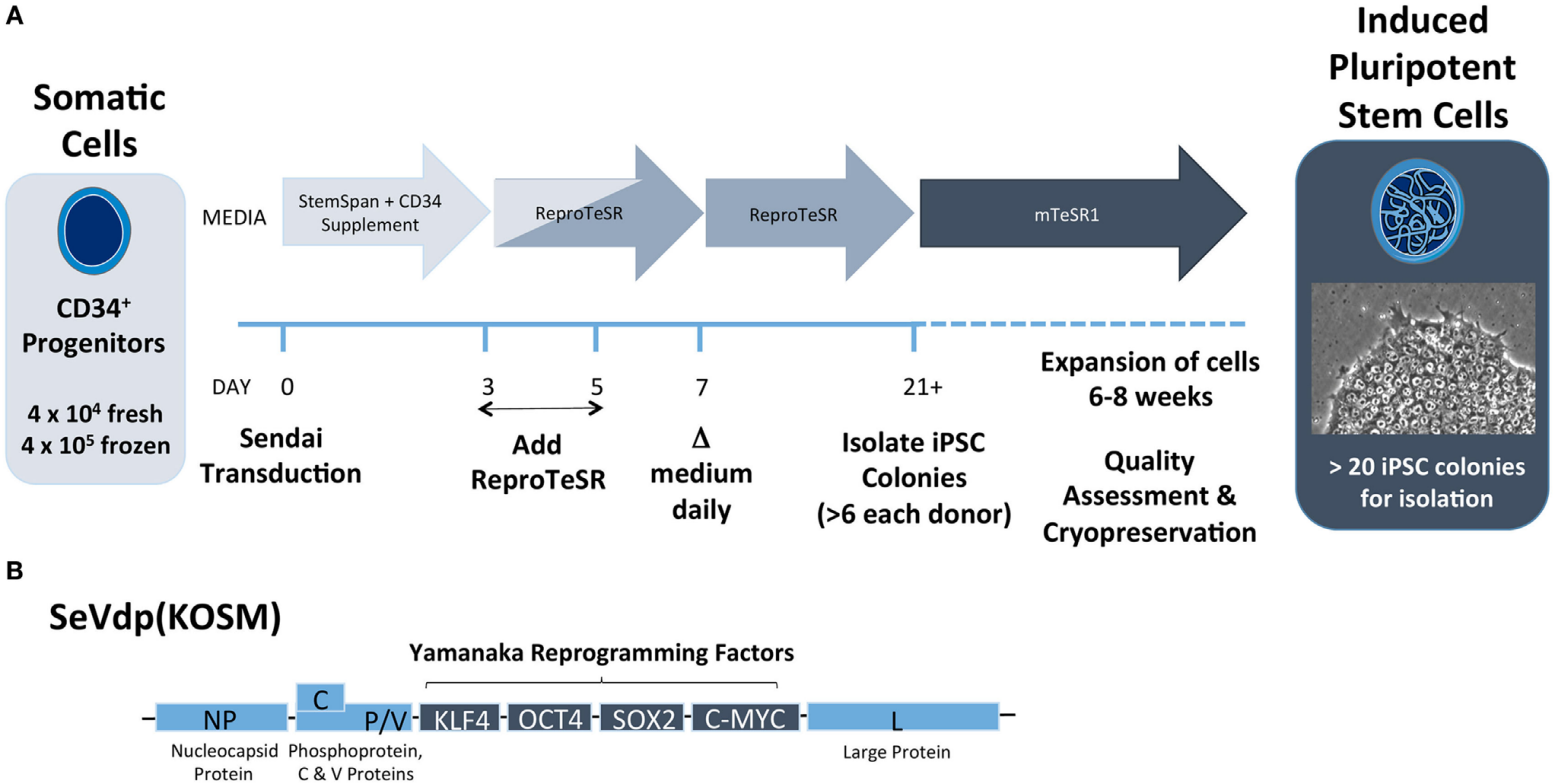
Induced pluripotent stem cell (iPSC) reprogramming schematic from CD34^+^ cells using Sendai virus. **(A)** CD34^+^ progenitor cells are transduced with Sendai virus on day 0 and are cultured in feeder-free conditions on Matrigel-coated plates with CD34^+^ expansion medium (e.g., StemSpan SFEMII plus StemSpan CD34^+^ Expansion Supplement) for the first 3 days. Medium to support reprogramming (e.g., ReproTeSR) is added on days 3 and 5. Starting on day 7, medium is replaced daily until colonies are sufficiently large to isolate clonally, typically on day 21. After isolation, cells are cultured in PSC maintenance medium (e.g., mTeSR1) for 4–6 weeks of expansion. After expansion, iPSCs undergo assessment for pluripotency and normal karyotype and can be cryopreserved for later use. **(B)** Replication defective Sendai virus (SeVdp KOSM) contains Sendai genes NP (nucleocapsid), P (Phosphoprotein), L (large protein), C protein, V protein, and Yamanaka reprogramming genes KLF4, OCT4, SOX2, and c-MYC.

Each investigator must determine starting cell source and reprogramming method based on available cells and the ultimate research plan. Our group has found that CD34^+^ HSCs isolated from peripheral blood can be efficiently reprogrammed into iPSCs using Sendai virus (Figure 5). This method has a few advantages over the use of PBMCs. First, CD34^+^ progenitor cells can be isolated from fresh, non-mobilized peripheral blood and expanded *in vitro* (111). Second, the efficiency to generate iPSCs is higher with this approach versus non-sorted PBMC. We have observed that as few as 2,000 isolated CD34^+^ HSCs yielded several iPSC colonies. Finally, the resultant iPSC will have native genetic configurations at both the TCR and immunoglobulin loci. However, CD34^+^ cells may not always be the best source of donor material. Where a re-arranged TCR with known antigen recognition is desired, CD4^+^ or CD8^+^ T cells from T1D patients could be used. In addition, it is known that T-cell-derived iPSCs differentiate back into T cells more efficiently, putatively due to epigenetic memory of the lineage (73).

In addition to cell type, it is particularly important to know the T1D risk genotype(s) of donor materials. One initiative at the University of Florida Center for Cellular Reprogramming is building an iPSC resource for genomic medicine. Samples from 50 healthy donors (25 males/25 females) with genome-wide SNP typing performed using the ImmunoChip platform are being utilized to generate iPSC lines. The SNP library will include all known T1D risk variants making this cell library and others like it (e.g., the Helmsley Cellular Research Hub, cellhub.org) powerful tools for studying complex genetic traits. Such an iPSC library with SNP database will provide an extremely useful common platform for SNP validation studies in combination with conventional gene editing technologies (Figure 4). For example, starting from iPSC clones harboring heterozygous status for a particular SNP (Figure 4, Module A), an investigator can obtain SNP hemizygous clones through CRISPR/Cas9-mediated allele targeting. Using such clones, one can study the effect of SNP variations within isogenic conditions in a relatively short timeframe.

Reprogramming somatic cells into iPSC is no longer limited to the investigators who have developed various methods in their own labs. Since the initial discovery of the “Yamanaka Factors” in 2006 where four minimal genes (*Oct3/4, Sox2, c-Myc*, and *Klf4*) were identified as key iPSC reprogramming factors (112), numerous advancements in reprogramming gene delivery have been made: these include delivery of reprogramming genes as lentiviral transgenes, plasmid DNA, or messenger RNA. Each of these platforms has become commercially available in reprogramming kits so that most labs can reprogram iPSC from a variety of tissues. Our group has found most success with a Sendai virus reprogramming vector. This non-integrating and self-limiting murine parainfluenza virus delivers the four essential iPSC genes in a single polycistronic message (Figure 5B) (113–115). Regardless of the method used, iPSCs take on a highly pluripotent phenotype and can be used to differentiate numerous lineages.

We have highlighted earlier progress in differentiating iPSC into key immune, endocrine, and endothelial cell types. Without doubt, future applications of this approach will continue to expand as the community derives additional cell types from iPSC progenitor populations. The ability to switch between protective and susceptible variants and effectively turn genes on/off or up/down will allow the reductionist types of mechanistic studies previously only possible in gene knockout or transgenic animal models. One can certainly envision future models employing iPSC that layer increasingly complex admixtures of cells to recapitulate tissue micro-environments complete with multiple endocrine cell types, acinar tissues, microvasculature, and perhaps even innervation. We, along with others, are beginning to print living cells into liquid-like solid matrices allowing for exquisite control of cellular distribution in 3D space (116). The preliminary transcriptional profiles that have emerged from the transition from 2D culture in plastic wells to 3D cell culture have already suggested a distinct gene expression signature, more akin to that extracted from native tissues. Specific investigations using such 3D culture systems together with isogenic cellular models are needed to examine T1-IFN signaling with modulation of IFIH1 and TYK2 risk alleles as well as costimulatory pathways known to confer T1D risk (e.g., CD28/CTLA4 and CD226/TIGIT) (Table 1). Moreover, there will certainly be applications to reconstruct immune developmental niches to recapitulate key elements of hematopoietic development in the bone marrow, thymus, and secondary lymphoid organs. Such studies are expected to afford novel drug discovery through identification of new therapeutic targets.

The most obvious applications for stem cells in the T1D field reside in the ongoing need to replace the loss and functional inactivity of endogenous β cell mass that precipitates glucose dysregulation [reviewed in Ref. (117)]. To date, this has been accomplished through both hES- and iPSC-derived insulin-producing β cells. The capacity to model and recreate not only β cells but also functional immune populations will allow the testing of therapies to close the translational loop and prevent recurrent auto- or allo-immune rejection of transplanted β cells. Indeed, this might be accomplished by introducing genes to protect iPSC-generated β cells against apoptosis (e.g., GLIS3) (118) or to shield them from immunological attack, representing key objectives for iPSC-derived treatments in the regenerative medicine space.

Interventional trials to restore or preserve β cells in T1D have largely been driven by individual investigator sponsored trials in the context of larger consortiums (e.g., TrialNet and the Immune Tolerance Network). These efforts have largely taken the form of repurposing clinically approved drugs from other diseases or have been based on preliminary studies generated in the NOD mouse model of T1D. While these efforts are beginning to demonstrate some transient preservation of C-peptide (the serum marker co-secreted in equimolar amounts with insulin), no current therapy has yet resulted in an FDA-approved intervention capable of demonstrating long-term efficacy (119–128). We propose that additional dose finding studies using human isogenic cellular systems to screen for desired mechanistic outcomes could potentially inform clinical trial agent selection and dosing.

From a patient perspective, the notion of equipoise limits experimental testing of many novel and/or high-risk combinatorial agents. By adopting isogenic cellular systems, those limitations could be mitigated by testing and optimizing prior to trial validations. Moreover, despite some demonstration of efficacy in preliminary trials (e.g., teplizumab, abatacept, alefacept, and ATG with or without G-CSF), no clear marker has emerged *a priori* that effectively predicts clinical responders or non-responders to any particular agent beyond basic cohort demographics of age, residual C-peptide, and disease duration (127–129). The use of isogenic cellular systems and personalized testing could facilitate drug selection and dose optimizations with clearly defined mechanistic readouts (e.g., phosphorylated-STAT5 response following low-dose IL-2) (130–133). Ultimately, the advent of genomic editing and isogenic cellular systems will not only enable a deeper understanding of disease pathogenesis but should also expedite the speed of discovery and clinical translation with the hope of both restoring β cell mass and inducing durable antigen-specific immunological tolerance.

## Conclusion

The emergence of genomic medicine has accelerated the rate of discovery with regard to the genetic basis of T1D. Multidimensional datasets now make it possible to overlay components of genetic variation, epigenetics, and transcriptional control of gene expression. Unfortunately, the vast number of associated SNPs, heterogeneity in human disease, and limits of clinical resources present a new set of challenges. There remains a paramount need to move beyond discovery of associated SNPs to a deeper understanding of causative variants to elucidate the molecular mechanisms and pathways of disease. The advent of iPSC technologies and precision gene editing now allows researchers to expedite the discovery and validation of these disease-associated variants.

Induced pluripotent stem cell technologies were initially met with great enthusiasm with the prospect of offering the capacity for regenerative medicine applications, including autologous β cell replacement in T1D. While the robustness and efficiency of these approaches will continue to advance, the current technologies exist to derive these cells, enabling researchers to build more powerful models of disease pathogenesis. Specifically, isogenic cellular systems now allow modeling of target β cells, effector T-cell populations, and the innate and stromal components that interact with both the target organ and effector arms of the immune system. The capacity to rapidly derive these highly limited and rare populations at scale, all while targeting genomic loci in a high-throughput manner is expected to expedite functional genomics in a manner heretofore not observed. A detailed understanding of the mechanisms by which gene variants confer susceptibility or protection to disease will undoubtedly identify a number of key immunological lynchpins that can be therapeutically targeted in a rational approach to restore immune tolerance to β cells in individuals with T1D.

## Author Contributions

MW and TB conceived the content and contributed to the writing and editing of the manuscript. KS and NT contributed to the writing and editing of the manuscript.

## Conflict of Interest Statement

The authors declare that the research was conducted in the absence of any commercial or financial relationships that could be construed as a potential conflict of interest.
